# A Platform for Designing Genome-Based Personalized Immunotherapy or Vaccine against Cancer

**DOI:** 10.1371/journal.pone.0166372

**Published:** 2016-11-10

**Authors:** Sudheer Gupta, Kumardeep Chaudhary, Sandeep Kumar Dhanda, Rahul Kumar, Shailesh Kumar, Manika Sehgal, Gandharva Nagpal, Gajendra P. S. Raghava

**Affiliations:** Bioinformatics Centre, CSIR-Institute of Microbial Technology, Chandigarh-160036, India; Nazarbayev University, KAZAKHSTAN

## Abstract

Due to advancement in sequencing technology, genomes of thousands of cancer tissues or cell-lines have been sequenced. Identification of cancer-specific epitopes or neoepitopes from cancer genomes is one of the major challenges in the field of immunotherapy or vaccine development. This paper describes a platform Cancertope, developed for designing genome-based immunotherapy or vaccine against a cancer cell. Broadly, the integrated resources on this platform are apportioned into three precise sections. First section explains a cancer-specific database of neoepitopes generated from genome of 905 cancer cell lines. This database harbors wide range of epitopes (e.g., B-cell, CD8^+^ T-cell, HLA class I, HLA class II) against 60 cancer-specific vaccine antigens. Second section describes a partially personalized module developed for predicting potential neoepitopes against a user-specific cancer genome. Finally, we describe a fully personalized module developed for identification of neoepitopes from genomes of cancerous and healthy cells of a cancer-patient. In order to assist the scientific community, wide range of tools are incorporated in this platform that includes screening of epitopes against human reference proteome (http://www.imtech.res.in/raghava/cancertope/).

## Introduction

Worldwide, cancer is one of the most prominent cause of immature deaths every year [1]. In addition to millions of deaths each year, all countries are spending billions of dollars on treatment of cancer patients. In past, effective vaccines have been developed successfully against number of frightening diseases (e.g. small pox, polio); saving millions of lives. Subsequently, it is extremely important to develop effective vaccines against cancer to protect the human population from this awful disease. In this direction, researchers have got limited success in designing vaccine against cancers particularly against cancer-inducing viruses [2,3]. There are a number of hurdles in developing cancer vaccines that includes cross-reactivity, tolerance and insufficient immune response [4]. Similarly, the identification of mutations shared across wide range of cancer patients is also a challenge [5,6]. However, with advent of high throughput sequencing and assay techniques, different authors have made an attempt to investigate important shared mutations in various types of cancers [7,8]. Furthermore, in order to design a successful vaccine, it is important to identify cancer-specific antigens or antigenic regions that can induce immune system specifically against cancerous cells. These antigens and antigenic regions are called neoantigens and neoepitopes respectively. In past, number of experimental techniques has been developed to identify vaccine candidates (e.g., neoantigens, neoepitopes) for designing cancer vaccines [9,10].

Although there are reports of identification of vaccine candidates at genome scale, but the task is demanding because experimental techniques are costlier and time consuming with large amount of samples. In order to overcome the limitations of experimental techniques, numerous computational tools have been developed for designing vaccines or immunotherapy against cancer. Broadly, these computational tools can be divided in two categories: i) methods for predicting epitopes, and ii) prediction of potential vaccine candidates for cancer. In past, numerous direct or indirect epitope predictions have been developed for predicting antigenic regions that can activate B-cell, T-helper and cytotoxic T-cells [11,12]. In case of prediction of cancer vaccine targets, first cancer-specific regions are identified and then their immunogenic properties are predicted. Warren *et al*. (2010) identified mutated regions in antigens/proteins generated due to somatic mutations (missense, frame shift, insertion, and deletion) in human tumors [11]. They predicted HLA class I binders in these mutated regions and identified 159 potential vaccine candidates. Similarly, Khalili *et al*. (2012) predicted HLA-A and B binders in mutated region of 312 genes; generated due to missense mutations [13]. Brown *et al*. identified immunogenic mutations in the form of HLA class I binders from sequencing data of 515 patients [14]. In this study, authors endeavored to correlate the presence of immunogenic missense mutations with the survival of patients. Recently, Rajasagi *et al*. proposed 22 HLA class I binders generated from missense mutations through a developed pipeline for 91 chronic lymphocytic leukemias [15]. In most of the above studies, authors predicted only HLA class I binders or cytotoxic T-cell (CTL) epitopes.

There are several computational tools for the prediction of HLA binding peptides and T-cell epitopes and B cell epitopes, which can be used for the prediction of immunogenic mutated regions in an antigen. However, there is a necessity for a streamlined computational tool that allows users to identify immunogenic mutations and the predicted cancer epitopes. One of the major limitations of existing computational tools for predicting cancer vaccine candidates is that they do not predict B-cell or T-helper epitopes. In addition, there is no specific computation resource for predicted cancer epitopes in user-specified genome. Aim of this study is complementing existing methods and to address unresolved issues. We analyzed mutational profile of 905-cancer cell lines and identified neoepitopes that can activate different arms of immune system. This information has been compiled in the form of a database so that the user can access cancer-specific epitopes for any cancer cell line. In addition, fully and partially personalized pipelines have been integrated in this database to facilitate scientific community. In brief, the study illustrates exclusive evaluation of immune epitopes on the mutational landscape of a large number of cancer cell lines (https://figshare.com/articles/CANCERTOPE_MUTATION_DATASET_txt/4176558) and eventually postulates a workbench, named Cancertope for designing neoepitope-based personalized vaccines/immunotherapies (http://crdd.osdd.net/raghava/cancertope/).

## Results

### Analysis of Vaccine Targets

**Fig 1:**
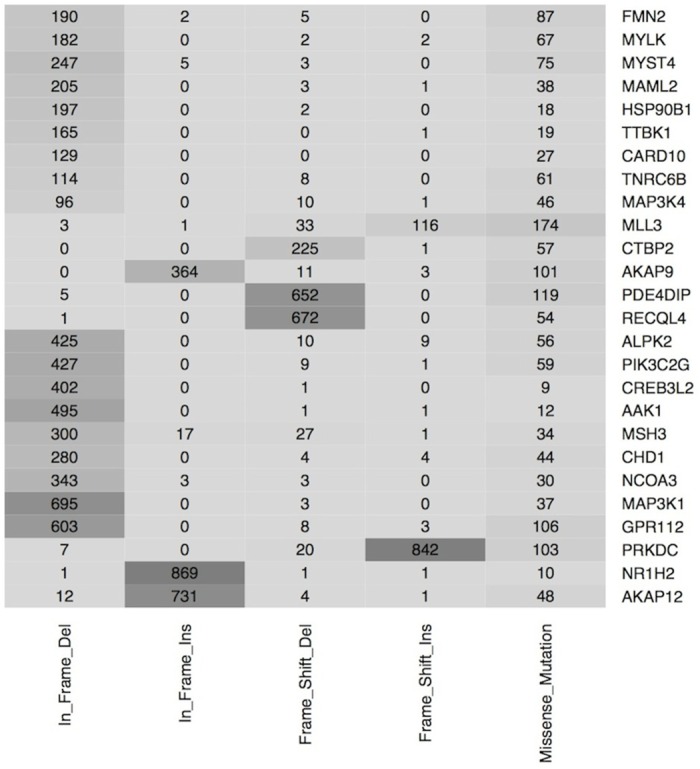
Frequency and type of mutations reported for each vaccine candidate. Each numerical value representing the number of mutations across different cell lines in a vaccine candidate, for instance, vaccine target PRKDC has been mutated 842 times (frame shift insertions) in the different cell lines.

The current study is based on 60 vaccine candidates, 26 reported from the analysis of NGS data from CCLE database [16] and remaining 34 candidates from CanProVar [17] based on their association with cancer. The 26 genes (vaccine candidates) were selected from CCLE as they frequently mutate in different types of cell lines (see Methods section). The distribution and types of mutations were then analyzed in vaccine candidates, which further depicted the prominence of missense mutation type (Fig 1). Similarly, the frame shift mutations in a few key genes like *PRKDC*, *RECQL4*, *PDE4DIP*, and *CTBP2* were found in harmony with a large number of cell lines. Also, the in-frame insertions and deletions were very profound in genes like *AKAP12*, *NR1H2*, *GPR112*, and *MAP3K1*. All these genes in the study are being referred to as cancer sensitive genes since they possess higher probability to be associated with cancer on encountering mutations. In other words, a gene is called cancer-sensitive, if the mutations in that gene have high propensity of being cancer-associated.

**Fig 2:**
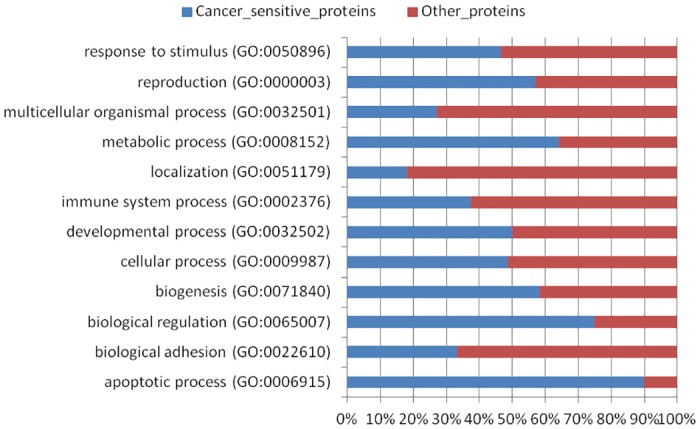
The functional characterization of cancer-sensitive and other proteins based on their gene ontologies.

Furthermore, Table 1 presents 34 vaccine targets possessing mutations that exhibit higher probability of transforming a normal cell into a cancerous cell as selected from CanProVar. Among these vaccine candidates, mutations in targets like PTEN [18], TP53 [18], BRAF [19], EGFR [20] and c-KIT [21,22] have already been reported in earlier studies to be highly carcinogenic and proposed to be targeted for intending immunotherapies. These analyses support our criteria of selection of generalized vaccine candidates. To further broaden the perspective of functional analysis, the cancer sensitive genes were compared with all other genes on the basis of their gene ontologies. The analyses uncovered interesting observations suggesting involvement of cancer sensitive proteins is somehow greater in the apoptotic processes, biological regulation, catalytic and binding activities as compared to the other proteins (Fig 2 and S1 Fig).

**Table 1:** Number of deleterious mutations (f_D_), polymorphism/neutral variants (f_P_) and cancer association (f_D_/f_P_) in each vaccine target.

Target	f_D_	f_P_	f_D_/f_P_	Family/subfamily of target/protein
PTEN	389	1	389	NA
TP53	1353	7	193.3	P53_family
CTNNB1	132	1	132	Beta-catenin_family
BRAF	99	1	99	Protein_kinase_superfamily,_TKL_Ser/Thr_protein_kinase_family,_RAF_subfamily
NF2	74	1	74	NA
EGFR	188	3	62.7	Protein_kinase_superfamily,_Tyr_protein_kinase_family,_EGF_receptor_subfamily
SMAD4	107	2	53.5	Dwarfin/SMAD_family
VHL	272	6	45.3	NA
KIT	131	3	43.7	Protein_kinase_superfamily,_Tyr_protein_kinase_family,_CSF-1/PDGF_receptor_subfamily
PIK3CA	174	4	43.5	PI3/PI4-kinase_family
NRAS	36	1	36	Small_GTPase_superfamily,_Ras_family
MSH2	103	5	20.6	DNA_mismatch_repair_MutS_family
GATA1	20	1	20	NA
MLH1	118	6	19.7	DNA_mismatch_repair_MutL/HexB_family
FBXW7	67	4	16.8	NA
MEN1	49	3	16.3	NA
FGFR3	31	2	15.5	Protein_kinase_superfamily,_Tyr_protein_kinase_family,_Fibroblast_growth_factor_receptor_subfamily
TSHR	46	3	15.3	G-protein_coupled_receptor_1_family,_FSH/LSH/TSH_subfamily
JAK2	40	3	13.3	Protein_kinase_superfamily,_Tyr_protein_kinase_family,_JAK_subfamily
RB1	102	8	12.8	Retinoblastoma_protein_(RB)_family
PDGFRA	35	3	11.7	Protein_kinase_superfamily,_Tyr_protein_kinase_family,_CSF-1/PDGF_receptor_subfamily
NF1	65	6	10.8	NA
FGFR2	43	4	10.8	Protein_kinase_superfamily,_Tyr_protein_kinase_family,_Fibroblast_growth_factor_receptor_subfamily
FLT3	35	4	8.8	Protein_kinase_superfamily,_Tyr_protein_kinase_family,_CSF-1/PDGF_receptor_subfamily
CDH1	68	8	8.5	NA
TNFAIP3	31	4	7.8	Peptidase_C64_family
CBL	30	4	7.5	NA
RET	58	8	7.3	Protein_kinase_superfamily,_Tyr_protein_kinase_family
MSH6	40	8	5	DNA_mismatch_repair_MutS_family
ERBB2	29	6	4.8	Protein_kinase_superfamily,_Tyr_protein_kinase_family,_EGF_receptor_subfamily
MET	23	5	4.6	Protein_kinase_superfamily,_Tyr_protein_kinase_family
ABL1	23	7	3.3	Protein_kinase_superfamily,_Tyr_protein_kinase_family,_ABL_subfamily
ALK	27	9	3	Protein_kinase_superfamily,_Tyr_protein_kinase_family,_Insulin_receptor_subfamily
ATM	134	52	2.6	PI3/PI4-kinase_family,_ATM_subfamily

### Expression Analysis of Cancer Vaccine Candidates

As stated earlier, cancer vaccine candidates were selected on the basis of their mutation frequency in cancer cell lines and their level of association with cancer. Next, the expression profile of these genes was examined in all available cancer cell lines. As displayed in Table 2, most of the vaccine candidates were highly expressed in a large number of cell lines. Since, the attained expression data ranged from 2 to 15, the expression values were randomly divided into four bins for well-defined understanding and the genes with expression values > = 9 were anticipated as highly expressed genes. With this assumption, it was perceived that the candidate genes i.e. *HSP90B1*, *MLH1*, *MSH6*, *PRKDC*, *MSH2*, and *AKAP9* are highly expressed in more than 700 cell lines.

**Table 2:** Expression analysis depicting number of cell lines with expression more than a given cutoff (e.g., 3, 7, 9) for each antigen.

Target	>= 3	>= 7	>= 9	>= 12
AAK1	901	0	0	0
ABL1	901	900	595	0
AKAP12	901	509	294	8
AKAP9	901	900	723	2
ALK	901	23	12	1
ALPK2	901	179	92	1
ATM	901	816	165	0
BRAF	901	282	1	0
CARD10	901	591	109	0
CBL	901	754	4	0
CDH1	901	358	217	0
CHD1	901	901	644	0
CREB3L2	901	849	424	1
CTBP2	901	802	638	0
CTNNB1	901	807	60	0
EGFR	901	318	21	0
ERBB2	901	368	39	14
FBXW7	NA	NA	NA	NA
FGFR2	901	164	34	1
FGFR3	901	88	6	0
FLT3	901	35	27	3
FMN2	901	101	22	0
GATA1	901	19	17	0
GPR112	901	0	0	0
HSP90B1	901	901	897	282
JAK2	901	68	7	1
KIT	901	170	82	12
MAML2	901	49	0	0
MAP3K1	901	393	28	0
MAP3K4	901	900	678	0
MEN1	NA	NA	NA	NA
MET	NA	NA	NA	NA
MLH1	901	875	861	2
MLL3	0	0	0	0
MSH2	901	887	731	0
MSH3	901	555	1	0
MSH6	901	892	820	7
MYLK	901	473	286	26
MYST4	NA	NA	NA	NA
NCOA3	901	825	156	0
NF1	901	264	2	0
NF2	901	19	0	0
NR1H2	901	161	0	0
NRAS	901	891	675	4
PDE4DIP	901	198	12	0
PDGFRA	901	115	73	9
PIK3C2G	901	14	2	0
PIK3CA	901	856	89	0
PRKDC	901	901	815	3
PTEN	901	834	315	0
RB1	901	674	26	0
RECQL4	901	804	39	0
RET	901	52	15	0
SMAD4	901	575	5	0
TNFAIP3	901	596	252	9
TNRC6B	901	803	8	0
TP53	901	597	75	0
TSHR	901	22	3	0
TTBK1	901	0	0	0
VHL	901	499	5	0

For example, *HSP90B1* has 282 cell lines having expression greater or equal to 12.

### Identification of Neopeptides

After scrutinizing 60 potential vaccine candidates, the next challenge was to identify cancer-specific regions/peptides in these vaccine candidates. Therefore, overlapping 9-mer peptides for each of the vaccine candidates (Table 3) were created and different filters were applied in order to identify cancer-specific peptides generated due to cancer-associated mutations. These filters refined the dataset by eliminating all those peptides whose identical sequence maps to the genome of healthy individuals. The criteria adopted for removing identical peptides focused on i) reference protein, 2) reference proteome, 3) 1000 Genomes-based variants of the same antigen and 4) 1000 Genomes-based proteomes. It was observed that the candidates such as TP53, MLL3, PDE4DIP, PRKDC and certain others have the highest number of unique neopeptides, not present in reference proteome or 1000 Genomes-based proteomes.

**Table 3:** Total number of generated neopeptides (9-mer peptides) in each vaccine candidate and number of neopeptides after applying different filters.

Vaccine Candidate	Total 9-mer	Reference Protein	Reference Proteome	1000-Genome Proteomes
TP53	2589	2204	2204	2204
MLL3	6570	1671	1671	1670
PDE4DIP	3468	1130	1121	1013
PRKDC	5269	1149	1149	1149
TNRC6B	2730	905	905	886
AKAP9	4873	974	974	938
ATM	4016	968	968	968
GPR112	4061	989	989	989
FMN2	2322	850	805	797
NF1	3650	819	819	810
MYST4	2705	668	668	639
PTEN	1148	753	753	753
CTBP2	1550	573	573	573
ALK	2185	573	573	557
MYLK	2512	615	615	596
ALPK2	2765	603	603	603
AKAP12	2265	491	491	430
MAML2	1536	443	443	440
MAP3K4	2078	482	482	480
PIK3CA	1573	513	513	513
SMAD4	1005	461	461	460
RECQL4	1658	458	458	431
PDGFRA	1563	482	473	461
MSH6	1839	487	487	459
CHD1	2209	507	507	490
PIK3C2G	1881	444	426	418
CDH1	1279	405	405	405
EGFR	1628	426	426	426
FGFR3	1190	390	390	380
MSH3	1489	363	363	334
FBXW7	1111	412	412	409
MET	1813	413	413	401
TNFAIP3	1139	357	357	348
CTNNB1	1122	349	349	349
RB1	1241	321	321	321
RET	1459	353	353	353
NCOA3	1701	305	305	305
KIT	1280	312	312	303
MLH1	1063	315	315	306
MAP3K1	1835	331	330	322
BRAF	1106	348	348	345
FLT3	1290	305	305	295
FGFR2	1102	288	288	288
ABL1	1400	259	259	259
JAK2	1379	255	255	255
MSH2	1198	272	272	254
CARD10	1264	241	241	232
TSHR	1017	261	261	261
ERBB2	1482	235	235	232
CBL	1123	225	225	223
NRAS	380	199	199	189
MEN1	804	197	197	197
TTBK1	1483	189	189	180
NF2	798	211	211	201
GATA1	569	164	164	164
HSP90B1	980	185	185	185
CREB3L2	670	158	158	158
NR1H2	592	139	139	138
AAK1	1080	132	132	122
VHL	297	92	92	92

The filters remove neoepitopes present in reference protein, human reference proteome and 1000 Genomes-based proteomes.

### Evaluating Neopeptides as Neoepitopes

The generated neopeptides in the study were further analyzed for their roles as neoepitopes, i.e. antigenic region of nine amino acids specifically found in cancer antigens that can substantially activate different arms of the human immune system. In order to identify neoepitopes, different prediction tools were used for estimation of distinct epitopes [23,24,25,26,27]. Among all the tissue of origins, cell lines were explored for tissue-specific neoepitopes. Most frequent (top 10) neoepitopes along with their immunological potential are shown in the S1 Table. Interestingly, “IRKQQQQQE” neoepitope, which was generated de novo because of mutation in NR1H2 protein, was frequently observed in hematopoietic, lung, kidney, biliary tract, CNS bone, ovary, pancreas, prostate and large intestine tissues related cell lines. Moreover, it also harbors B cell epitope and is a binder for MHC I, MHC II. Similarly, mutation in same gene and cell lines generated “QQQQQESQS” which is a B cell epitope. Furthermore, in case of solid tumors like large intestine, the total number of neoepitopes was the highest in MLL3 and PDE4DIP targets whereas for hematopoietic tumors, TP53 and PDE4DIP were found to have the highest number of neoepitopes (S2 Table). The analysis of 60 vaccine candidates provided 38 promiscuous epitopes that have the ability to induce all arms of the immune system (S3 Table). Additionally, there were interesting outcomes from each individual algorithm of our pipeline that has been complied in the resource. For example, PRKDC has 5 or more positive neoepitopes predicted using CTLPred and nHLAPred, which were present in more than 800 unique cell lines (S4 and S5 Tables). Also, there were more than 15 neopeptides found to be HLA class I binders (using ProPred1) from RECQL4 and PRKDC, which were present in more than 600 cell lines (S6 Table). Similarly, in case of HLA class II binders (ProPred), PDE4DIP has 7 or more neoepitopes (HLA class II), which were found in 184 cell lines (S7 Table). It was also found that there were 5 or more neoepitopes predicted to be positive using BCE from NR1H2, which were present in 868 cell linesrespectively (S8 Table).

### Web-Based *In Silico* Platform

Based on the extensive evaluation of cancer neoepitopes, an *in silico* platform, Cancertope, has been developed for guiding subunit-based vaccine development, immunotherapies and other therapeutic interventions. The resource offers potential vaccine candidates and antigenic regions or epitopes, suitable for designing subunit vaccines against cancer. This web-based platform has been developed on LAMP system (Linux, Apache, MySQL, and PHP/Perl). The webserver has integrated following modules in the platform for providing valuable insights into personalized cancer immunotherapies.

### Database of Neoepitopes

**Fig 3:**
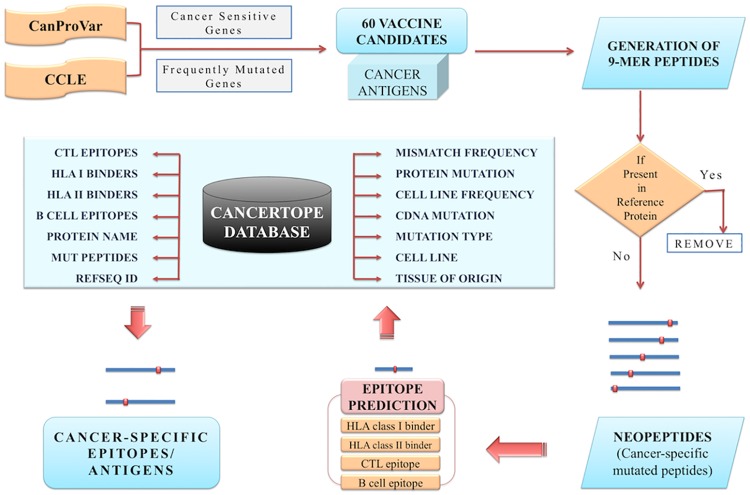
A general workflow exhibiting the overall concept of database section of Cancertope workbench.

The database consists of the analyses carried out on 905 human cancer cell lines, where a large number of immunogenic (neoepitopes) and non-immunogenic neopeptides is reported. The mutation and immune epitope information of cancer vaccine targets has been compiled in the form of ‘Cancer-specific database’ (Fig 3). For governing the effective utilization of the database, a number of standard database tools have been integrated for easy searching, browsing and retrieval of data.

### Partially Personalized Module

**Fig 4:**
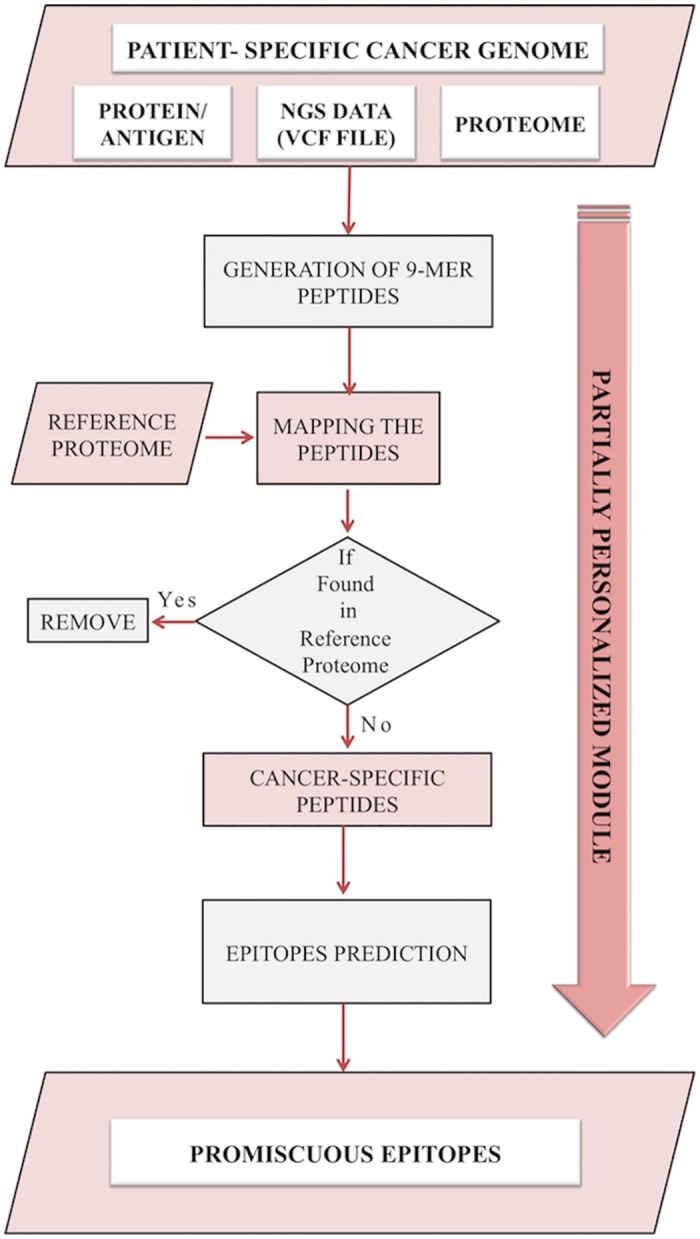
The personalized module of Cancertope workbench.

This module allows user to identify potential neoepitopes for designing vaccine against a cancer cell line and tissue of a sample from their genomic data. The term partially personalized is used to describe a situation, where the query sequence (from cancer tissue of a sample) is compared with the human reference proteome in the absence of normal/healthy (from non-cancerous tissue) proteome of that particular individual. This module compares user-specified cancer proteome with reference proteome and identifies potential neoepitopes (Fig 4). The module allows the user to submit a single protein sequence, whole proteome or VCF file from whole genome sequencing. The server will provide output in the form of potential neoepitopes.

### Fully Personalized Module

**Fig 5:**
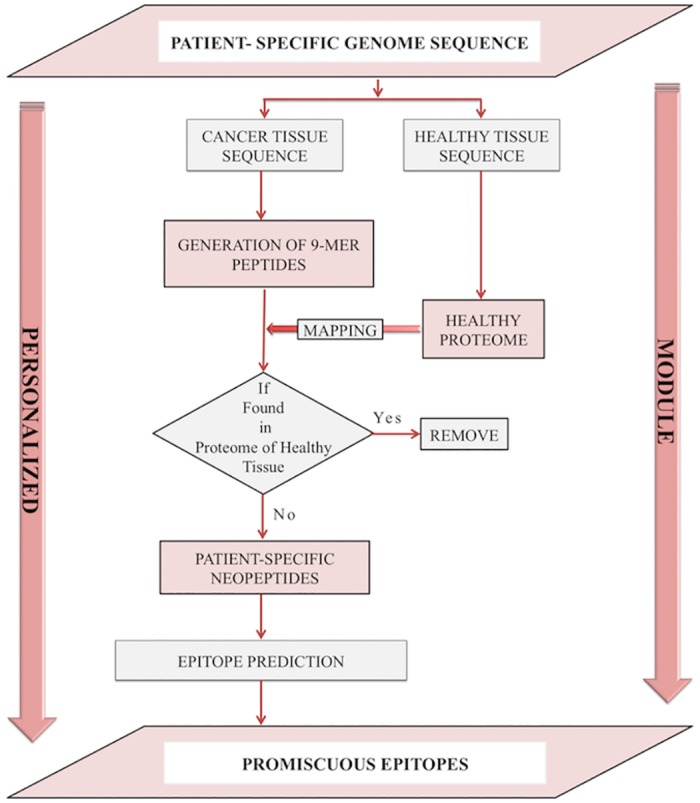
The fully personalized module of Cancertope workbench.

This module is designed for the identification of potential neoepitope-based vaccine candidates from proteomics data of cancerous and healthy tissues of a patient. User needs to provide protein or proteome of cancerous cells (or tissues) as well as of normal cells (healthy tissue) from the same individual (Fig 5). It will identify neopeptides and neoepitopes present in the proteome of cancer tissue but absent in proteome of healthy tissues. Like the partially personalized module, this module also allows the user to submit a pair of protein sequences, a pair of whole proteomes or VCF files from whole genome sequencing.

#### Advanced tools

This module provides two menus: i) Epitope Mapping for mapping experimentally validated epitopes, and ii) Cross-Reactivity for identification of cancer-specific peptides or neopeptides. ‘Epitope Mapping’ menu of Cancertope allows the user to identify antigenic regions in their protein sequence. In order to identify antigenic regions, we searched experimentally validated epitopes (e.g., B-cell, T-cell, HLA binders) present in major immunological databases like IEDB [28], MHCBN [29], BCIPEP [30]. ‘Cross-Reactivity’ menu is designed for removing neopeptides that are present specifically in cancer antigen submitted by the user and not in the human genome, in order to remove cross-reactive peptides. This ‘Cross-Reactivity’ menu expands the utility of the platform by allowing the user to search their antigen sequence against reference protein, human reference proteome and 1000 Genomes-based proteome.

## Discussion

Although the field of personalized cancer vaccine design using patient’s genomics data is in very primitive stages, the approach adopted for developing Cancertope suggests clinical as well as diagnostic potential. Since ages, cancer immunotherapy and vaccine development are being practiced as effective measures of therapeutic interventions. In 1999, Brossart *et al*. proved the potential implication of HLA-A2 restricted peptides in cancer therapies [31]. Although substantial growth in understanding of cancer induced by viruses such as papilloma virus and hepatitis B virus is achieved, but till date there is no significant success in the development of vaccines against these cancers. The difficulty in developing these vaccines is tolerance against self-antigens, risk of autoimmunity and heterogeneity in genomics of different cancers [32,33]. Cancertope provides well-defined filters that possess great significance in terms of cross reactivity by eliminating epitopes located in reference protein, human reference proteomeand 1000 Genomes-based proteomes. Thus, the provided filters assist in combating the pertaining concern of autoimmunity thus specifically activating immune system against cancer.

The use of cancer cell lines for immunological studies may be critical, since in absence of immunological pressure, the genomic profile of cancer cell lines may be ambiguous. However, this possibility has been ruled out by the correlation analysis preformed by CCLE study where the genomic similarities by lineage between CCLE cell lines and primary tumors from Tumorscape, expO, MILE and COSMIC data sets were inspected. The data from mutation frequencies in 17 lineages of CCLE and COSMIC primary tumor data revealed high correlation of these mutations with most of the lineages such as breast (r = 0.73), colorectal (r = 0.76), esophagus (r = 0.95), kidney (r = 0.85), liver (r = 0.64) and pancreas (r = 0.96). Since the mutational profile of cancer cell lines demonstrated significant correlation with patient tumor sample, therefore this sequence data was selected for the conducted immunological evaluation. The proposed vaccine candidates from Cancertope were highly expressed in most of the cell lines, which makes them suitable candidates because over expression is also considered as one of the prime criterion for developing cancer vaccines [34].

While, the immune epitope prediction tools used in this study were highly cited, published and accurate but still these prediction algorithms have their own limitations. Thus, the neoepitope/antigens should be experimentally validated before suggesting it for medical purpose. There are following major parameters which need to be tested to validate a neoepitope: (a) HLA binding of the peptide, (b) Display of the neoepitope on the tumor surface on MHC molecule (can be verified either by mass spectrometry or by using a T cell raised against the neoepitope), (c) Expression of the neoantigen in the tumor cells and (d) cross reactivity which means T cells against the peptide should not recognize the wild-type peptide. After considering these limitations, the applied strategy in the study will be beneficial for scientific community and pharmaceutical companies. The cancer genomics in combination with computational predictions and experimental validations of immune epitopes can be used for designing successful cancer vaccines for patients. A few commercialized agencies (http://neontherapeutics.com/, http://www.chordomafoundation.org/, http://www.vaccinogeninc.com/, http://gapvac.eu/ and http://www.epivax.com/) are already working in this direction.

The Cancertope resource delivers extensive information on cancer specific mutations and investigates the immunogenic potential of neoepitopes by employing several prediction algorithms. The database section of Cancertope stipulates all the generalized vaccine candidates that can be validated thus gearing cancer research. Additionally, the module dispensing insights into personalized vaccines (partially- and fully-personalized) for newly sequenced genome operates on the genome annotation. The annotation and immune prediction pipeline further suggests most effective vaccine candidates for the queried sequencing data. The resource also features additional options for experimental epitope mapping and removal of cross-reactive candidates valuable for determining suitable vaccine candidates.

## Conclusion

In summary, a web-based platform for predicting vaccine candidates effective against cancer is reported. The platform basically delivers two options to the users, i.e. database-specific and other being user-interactive prediction server. The database-specific service maintains neoepitopes examined in 905 cancer cell lines, which are key components for activating the immune system against cancer cell lines. Furthermore, the neoepitope-based database facilitates a demonstration for guiding the generation of neoepitopes against a tumor from its whole-genome. Although, the indicated cancer cell lines are correlated with patient tumor sample in genomic profiles yet the neoepitopes exemplified in our resource must be authorized experimentally before inclining them for clinical applications. For advancing the aim of personalized vaccine design against a patient or tissue-specific tumor, user-interactive interface has been designed by incorporating different modules. Under the user-interactive provision, server allows to identify cancer-specific epitopes against a tumor from its proteome/protein. In case, where user provides both healthy as well as tumor samples from the same patient, then the server’s personalized module identifies patient-specific potential neoepitopes. Further, these putative neoepitopes can then be targeted for designing vaccines and immunotherapies against cancer thus enabling personalized therapy in real life scenario. Although the prediction methods implemented in the Cancertope pipeline are highly accurate and cited by scientific community, the experimental validation and testing of parameters like HLA binding/expression of neoepitope, cross reactivity and T cell activation, is very important before going to clinical setup. However, the predicted vaccine candidates from Cancertope have higher potential to be experimentally authenticated because of their higher reported efficacies; consequently offering cost-effective, economical, timesaving and streamlined pipeline for acclaiming personalized cancer vaccines.

## Methods

### Source Data

The mutation profile of cancer cell lines was retrieved from Cancer Cell Line Encyclopedia (CCLE) [16] where MAF file was downloaded from data portal (http://www.broadinstitute.org/ccle/data/browseData). The selected dataset comprised the mutational profile of 1651 genes in 905 cell lines, where the variant filtration was done by exclusion of variants with low allelic fraction, common polymorphisms and putative neutral variants. Since the mutated protein sequences were not provided in CCLE database, the mutation profiles were mapped on to the reference cDNA sequences of each gene obtained from NCBI. Thereafter, the mutated cDNA of each gene was translated into mutant protein sequences. All the four types of mutations namely missense, frame shift, in-frame insertion and in-frame deletions were included in mutation profile.

### Selection of Cancer Vaccine Antigens

This section specifies the application of CanProVar (Cancer Proteome Variation) [17] database for selecting cancer vaccine candidates based on their cancer sensitivity. The database consists of single amino acid alterations in the human proteome and contains cancer-specific variations (cancer-sensitive mutations) and non-cancer specific variations in different proteins. First, the frequency of cancer-associated mutations (f_D_) and frequency of non-cancer specific variations (f_P_) for each protein, was computed. With a criteria of f_D_/f_P_> = 2 and f_D_> = 20, a total of 52 proteins were selected. These criteria were applied to select highly cancer sensitive proteins. Out of 52 proteins, only 34 proteins were found concurrent to CCLE study. These 34 proteins were then used as potential vaccine antigens or candidates and subsequently subjected to analyses via PANTHER classification system [35] (http://www.pantherdb.org/) to understand the properties of these antigens.

In addition, potential vaccine candidates were also identified from CCLE database based on their frequency of mutation. The mutational analysis revealed 26 proteins that were mutated in at least 10% (90 cell lines) of the cell lines. Finally, a total of 60 potential cancer vaccine candidates were obtained (34 cancer-associated antigens from CanProVar and 26 frequently mutated antigens from CCLE).

### Generation of Neopeptides

The term neopeptide in this study is being referred to the 9-mer sequences (9 residues continuous stretch of peptide) that contain at least one cancer-associated mutation. The length of neopeptide (epitope) was fixed to nine residues as both HLA class I and class II binders have a binding core of nine residues [36,37]. In order to identify neopeptides in a vaccine antigen, following steps were practiced: i) generated all possible overlapping peptides in an antigen, ii) removed redundant peptides and iii) removed all those peptides mapping to human reference proteome. This strategy expedited the detection of peptides exclusively present in the proteome of cancer cell lines but absent in proteome of a healthy individual.

### Pipeline for Predicting Immunogenicity

In order to estimate the immunogenicity of these neopeptides, a pipeline was established for prediction of different types of epitopes/binders. The pipeline integrated a number of algorithms for predicting diverse immune epitopes required for activating different arms of the immune system (CD4^+^ T cells, CD8^+^ T cells, B cells). The algorithms employed in the immune epitope prediction pipeline were preferred over other prevailing algorithms on the basis of availability in the standalone state. Moreover, the predictions from these algorithms have already been verified in a few experimental as well as *in silico* studies approving high accuracy and reliability of the softwares [38,39,40,41]. The immune epitope prediction can broadly be categorized into three categories.

### CD8^+^ T Cell Epitopes

In past, a number of methods have been reported for predicting HLA class I binders including SYFPEITHI [42], NetMHC [43], ProPred1 [24], and nHLAPred [25]. In the present study, we used standalone version of ProPred1 and nHLAPred for predicting HLA class I binders; both the algorithms predict promiscuous HLA class I binders. While, ProPred1 is a matrix-based method that predicts HLA binding sites in an antigenic sequence for 47 HLA class I alleles and nHLAPred was developed for envisaging 67 HLA class I binders using machine learning techniques. In addition to HLA class I binders as potential CTL epitopes, we also used a direct method, CTLPred, for predicting CTL epitopes. The prediction via direct method is critical as it discriminates between T cell epitopes and non-epitope MHC binders whereas HLA binding prediction only predicts the MHC binders from antigenic sequences.

### CD4^+^ T Cell Epitopes

Previously, a number of algorithms have been developed for predicting HLA class II binders such as ProPred [26], TEPITOPE [44] and NetMHCIIpan [45]. In this study, ProPred software has been used for predicting HLA class II binders. This software allows prediction of promiscuous HLA class II binders that can bind to a large number of alleles.

### B Cell Epitopes

There are numerous methods such as BCEPred [46], CBtope [47], LBtope [27], Discotope [48], COBEpro [49] available for predicting B-cell epitopes. We employed a standalone version of LBtope software for the prediction of linear B-cell epitopes. In order to predict immune epitopes in the query submitted by user at run time, all the prediction tools were required in standalone form. All the standalone prediction tools chosen for the study were heavily cited and were published in journals of high repute. The prediction standalones were used at default thresholds and parameters as optimized by the original authors.

#### Proteome data

In this study, the reference proteome and reference gene sequences were obtained from FTP portal of NCBI (http://ftp.ncbi.nlm.nih.gov/refseq/). In addition, the 1000 Genomes-based proteomes were generated by annotation of 1000 Genomes’ VCF files (http://ftp-trace.ncbi.nih.gov/1000genomes/ftp/release/20110521/) through ANNOVAR package [50]. The mutated sequence generation was done as mentioned in the ‘Source data’ section above.

#### Expression data

The expression profile of 905 cancer cell lines was obtained from CCLE database (http://www.ebi.ac.uk/arrayexpress/experiments/E-GEOD-36139/). In order to provide inclusive expression status of vaccine candidates, the number of cell lines with varying range of expressions were calculated; for instance > = 3 (GT3), > = 7 (GT7), > = 9 (GT9) and > = 12 (GT12); expression values ranging from 2–15.

## Supporting Information

S1 Fig The gene ontological information comprising of biological process, molecular function and cellular localization of cancer sensitive proteins. (TIFF)

S1 Table The top ten most frequent neopeptides for each tissue. For all the tissue of origin, most frequent neoepitopes were investigated and predicted for immune induction potential.
(XLSX)

S2 Table Representation of the number of neopeptides present in every tissue type. Each vaccine candidate is presented with number of unique neopeptide for each tissue of origin.
(XLSX)

S3 Table List of promiscuous neoepitopes with immunological potential in the form of CTL epitope, MHC binders, number of alleles, and B cell epitope. (XLSX)

S4 Table The number of cell lines having positive CTL epitopes in different range; for example PRKDC has 836 unique cell lines having total 5 or more unique CTL epitopes. The yellow cells present the number of neo-epitopes (CTL).
(XLSX)

S5 Table The number of cell lines having positive HLA I binders (ProPred1) in different range for example RECQL4 has 672 unique cell lines having total 15 or more unique HLA I binders. The yellow cells present the number of neo-epitopes (HLA I).
(XLSX)

S6 Table The number of cell lines having positive HLA I binders (nHLAPred) in different range for example PDE4DIP has 342 unique cell lines having total 7 or more unique HLA I binders. The yellow cells present the number of neo-epitopes (HLA I).
(XLSX)

S7 Table The number of cell lines having positive HLA II binders in different range for example PRKDC has 37 unique cell lines having total 5 or more unique HLA II binders. The yellow cells present the number of neo-epitopes (HLA II).
(XLSX)

S8 Table The number of cell lines having positive B cell epitopes in different range for example NR1H2 has 868 unique cell lines having total 5 or more unique B cell epitopes. The yellow cells present the number of neo-epitopes (BCE).
(XLSX)

## References

[1] Siegel RL, Miller KD, Jemal A (2016) Cancer statistics, 2016. CA Cancer J Clin 66: 7–30. 10.3322/caac.21332 26742998

[2] Morrow MP, Yan J, Sardesai NY (2013) Human papillomavirus therapeutic vaccines: targeting viral antigens as immunotherapy for precancerous disease and cancer. Expert Rev Vaccines 12: 271–283. 10.1586/erv.13.23 23496667

[3] Bergot AS, Kassianos A, Frazer IH, Mittal D (2011) New Approaches to Immunotherapy for HPV Associated Cancers. Cancers (Basel) 3: 3461–3495. 24212964 10.3390/cancers3033461 PMC3759206

[4] Igney FH, Krammer PH (2002) Immune escape of tumors: apoptosis resistance and tumor counterattack. J Leukoc Biol 71: 907–920. 12050175

[5] Fidler IJ (2012) Biological heterogeneity of cancer: implication to therapy. Hum Vaccin Immunother 8: 1141–1142. 10.4161/hv.19643 22854675 PMC3551889

[6] Fisher R, Pusztai L, Swanton C (2013) Cancer heterogeneity: implications for targeted therapeutics. Br J Cancer 108: 479–485. 10.1038/bjc.2012.581 23299535 PMC3593543

[7] Cai A, Keskin DB, DeLuca DS, Alonso A, Zhang W, et al (2012) Mutated BCR-ABL generates immunogenic T-cell epitopes in CML patients. Clin Cancer Res 18: 5761–5772. 10.1158/1078-0432.CCR-12-1182 22912393 PMC3759991

[8] Wang L, Lawrence MS, Wan Y, Stojanov P, Sougnez C, et al (2011) SF3B1 and other novel cancer genes in chronic lymphocytic leukemia. N Engl J Med 365: 2497–2506. 10.1056/NEJMoa1109016 22150006 PMC3685413

[9] Fisk B, Dague B, Seifert W, Kudelka A, Wharton J, et al (1997) Mass-spectrometric analysis of naturally processed peptides recognized by ovarian tumor-associated CD8(+) CTL. Int J Oncol 10: 159–169. 21533359 10.3892/ijo.10.1.159

[10] Schirle M, Keilholz W, Weber B, Gouttefangeas C, Dumrese T, et al (2000) Identification of tumor-associated MHC class I ligands by a novel T cell-independent approach. Eur J Immunol 30: 2216–2225. 10940913 10.1002/1521-4141(2000)30:8<2216::AID-IMMU2216>3.0.CO;2-7

[11] Warren RL, Holt RA (2010) A census of predicted mutational epitopes suitable for immunologic cancer control. Hum Immunol 71: 245–254. 10.1016/j.humimm.2009.12.007 20035814

[12] Dhanda SK, Usmani SS, Agrawal P, Nagpal G, Gautam A, et al (2016) Novel in silico tools for designing peptide-based subunit vaccines and immunotherapeutics. Brief Bioinform. 10.1093/bib/bbw025 27016393

[13] Khalili JS, Hanson RW, Szallasi Z (2012) In silico prediction of tumor antigens derived from functional missense mutations of the cancer gene census. Oncoimmunology 1: 1281–1289. 10.4161/onci.21511 23243591 PMC3518500

[14] Brown SD, Warren RL, Gibb EA, Martin SD, Spinelli JJ, et al (2014) Neo-antigens predicted by tumor genome meta-analysis correlate with increased patient survival. Genome Res 24: 743–750. 10.1101/gr.165985.113 24782321 PMC4009604

[15] Rajasagi M, Shukla SA, Fritsch EF, Keskin DB, DeLuca D, et al (2014) Systematic identification of personal tumor-specific neoantigens in chronic lymphocytic leukemia. Blood 124: 453–462. 10.1182/blood-2014-04-567933 24891321 PMC4102716

[16] Barretina J, Caponigro G, Stransky N, Venkatesan K, Margolin AA, et al (2012) The Cancer Cell Line Encyclopedia enables predictive modelling of anticancer drug sensitivity. Nature 483: 603–607. 10.1038/nature11003 22460905 PMC3320027

[17] Li J, Duncan DT, Zhang B (2010) CanProVar: a human cancer proteome variation database. Hum Mutat 31: 219–228. 10.1002/humu.21176 20052754 PMC2829365

[18] Castle JC, Kreiter S, Diekmann J, Lower M, van de Roemer N, et al (2012) Exploiting the mutanome for tumor vaccination. Cancer Res 72: 1081–1091. 10.1158/0008-5472.CAN-11-3722 22237626

[19] Somasundaram R, Swoboda R, Caputo L, Otvos L, Weber B, et al (2006) Human leukocyte antigen-A2-restricted CTL responses to mutated BRAF peptides in melanoma patients. Cancer Res 66: 3287–3293. 10.1158/0008-5472.CAN-05-1932 16540682

[20] Yamada T, Azuma K, Muta E, Kim J, Sugawara S, et al (2013) EGFR T790M mutation as a possible target for immunotherapy; identification of HLA-A*0201-restricted T cell epitopes derived from the EGFR T790M mutation. PLoS One 8: e78389 10.1371/journal.pone.0078389 24223798 PMC3818324

[21] Ashman LK, Griffith R (2013) Therapeutic targeting of c-KIT in cancer. Expert Opin Investig Drugs 22: 103–115. 10.1517/13543784.2013.740010 23127174

[22] Kato M, Takeda K, Kawamoto Y, Tsuzuki T, Hossain K, et al (2004) c-Kit-targeting immunotherapy for hereditary melanoma in a mouse model. Cancer Res 64: 801–806. 14871802 10.1158/0008-5472.can-03-2532

[23] Bhasin M, Raghava GP (2004) Prediction of CTL epitopes using QM, SVM and ANN techniques. Vaccine 22: 3195–3204. 10.1016/j.vaccine.2004.02.005 15297074

[24] Singh H, Raghava GP (2003) ProPred1: prediction of promiscuous MHC Class-I binding sites. Bioinformatics 19: 1009–1014. 12761064 10.1093/bioinformatics/btg108

[25] Bhasin M, Raghava GP (2007) A hybrid approach for predicting promiscuous MHC class I restricted T cell epitopes. J Biosci 32: 31–42. 17426378 10.1007/s12038-007-0004-5

[26] Singh H, Raghava GP (2001) ProPred: prediction of HLA-DR binding sites. Bioinformatics 17: 1236–1237. 11751237 10.1093/bioinformatics/17.12.1236

[27] Singh H, Ansari HR, Raghava GP (2013) Improved method for linear B-cell epitope prediction using antigen's primary sequence. PLoS One 8: e62216 10.1371/journal.pone.0062216 23667458 PMC3646881

[28] Zhang Q, Wang P, Kim Y, Haste-Andersen P, Beaver J, et al (2008) Immune epitope database analysis resource (IEDB-AR). Nucleic Acids Res 36: W513–518. 10.1093/nar/gkn254 18515843 PMC2447801

[29] Lata S, Bhasin M, Raghava GP (2009) MHCBN 4.0: A database of MHC/TAP binding peptides and T-cell epitopes. BMC Res Notes 2: 61 10.1186/1756-0500-2-61 19379493 PMC2679046

[30] Saha S, Bhasin M, Raghava GP (2005) Bcipep: a database of B-cell epitopes. BMC Genomics 6: 79 10.1186/1471-2164-6-79 15921533 PMC1173103

[31] Brossart P, Heinrich KS, Stuhler G, Behnke L, Reichardt VL, et al (1999) Identification of HLA-A2-restricted T-cell epitopes derived from the MUC1 tumor antigen for broadly applicable vaccine therapies. Blood 93: 4309–4317. 10361129

[32] Bodey B, Bodey B Jr., Siegel SE, Kaiser HE (2000) Failure of cancer vaccines: the significant limitations of this approach to immunotherapy. Anticancer Res 20: 2665–2676. 10953341

[33] Emens LA (2008) Cancer vaccines: on the threshold of success. Expert Opin Emerg Drugs 13: 295–308. 10.1517/14728214.13.2.295 18537522 PMC3086397

[34] Kwak LW (2011) Cancer vaccines: moving toward prevention? Cancer Prev Res (Phila) 4: 954–956. 21733818 10.1158/1940-6207.CAPR-11-0236

[35] Mi H, Muruganujan A, Casagrande JT, Thomas PD (2013) Large-scale gene function analysis with the PANTHER classification system. Nat Protoc 8: 1551–1566. 10.1038/nprot.2013.092 23868073 PMC6519453

[36] Rammensee HG, Friede T, Stevanoviic S (1995) MHC ligands and peptide motifs: first listing. Immunogenetics 41: 178–228. 7890324 10.1007/BF00172063

[37] Nielsen M, Lund O, Buus S, Lundegaard C (2010) MHC class II epitope predictive algorithms. Immunology 130: 319–328. 10.1111/j.1365-2567.2010.03268.x 20408898 PMC2913211

[38] Mustafa AS, Shaban FA (2006) ProPred analysis and experimental evaluation of promiscuous T-cell epitopes of three major secreted antigens of Mycobacterium tuberculosis. Tuberculosis (Edinb) 86: 115–124. 16039905 10.1016/j.tube.2005.05.001

[39] Lin HH, Zhang GL, Tongchusak S, Reinherz EL, Brusic V (2008) Evaluation of MHC-II peptide binding prediction servers: applications for vaccine research. BMC Bioinformatics 9 Suppl 12: S22. 10.1186/1471-2105-9-S12-S22 PMC2638162 19091022

[40] Mustafa AS (2011) Comparative evaluation of MPT83 (Rv2873) for T helper-1 cell reactivity and identification of HLA-promiscuous peptides in Mycobacterium bovis BCG-vaccinated healthy subjects. Clin Vaccine Immunol 18: 1752–1759. 10.1128/CVI.05260-11 21852544 PMC3187038

[41] Roider J, Meissner T, Kraut F, Vollbrecht T, Stirner R, et al (2014) Comparison of experimental fine-mapping to in silico prediction results of HIV-1 epitopes reveals ongoing need for mapping experiments. Immunology 143: 193–201. 10.1111/imm.12301 24724694 PMC4172136

[42] Schuler MM, Nastke MD, Stevanovikc S (2007) SYFPEITHI: database for searching and T-cell epitope prediction. Methods Mol Biol 409: 75–93. 18449993 10.1007/978-1-60327-118-9_5

[43] Lundegaard C, Lamberth K, Harndahl M, Buus S, Lund O, et al (2008) NetMHC-3.0: accurate web accessible predictions of human, mouse and monkey MHC class I affinities for peptides of length 8–11. Nucleic Acids Res 36: W509–512. 10.1093/nar/gkn202 18463140 PMC2447772

[44] Zhang L, Chen Y, Wong HS, Zhou S, Mamitsuka H, et al (2012) TEPITOPEpan: extending TEPITOPE for peptide binding prediction covering over 700 HLA-DR molecules. PLoS One 7: e30483 10.1371/journal.pone.0030483 22383964 PMC3285624

[45] Karosiene E, Rasmussen M, Blicher T, Lund O, Buus S, et al (2013) NetMHCIIpan-3.0, a common pan-specific MHC class II prediction method including all three human MHC class II isotypes, HLA-DR, HLA-DP and HLA-DQ. Immunogenetics 65: 711–724. 10.1007/s00251-013-0720-y 23900783 PMC3809066

[46] Saha S, Raghava GP (2007) Prediction methods for B-cell epitopes. Methods Mol Biol 409: 387–394. 10.1007/978-1-60327-118-9_29 18450017

[47] Ansari HR, Raghava GP (2010) Identification of conformational B-cell Epitopes in an antigen from its primary sequence. Immunome Res 6: 6 10.1186/1745-7580-6-6 20961417 PMC2974664

[48] Kringelum JV, Lundegaard C, Lund O, Nielsen M (2012) Reliable B cell epitope predictions: impacts of method development and improved benchmarking. PLoS Comput Biol 8: e1002829 10.1371/journal.pcbi.1002829 23300419 PMC3531324

[49] Sweredoski MJ, Baldi P (2009) COBEpro: a novel system for predicting continuous B-cell epitopes. Protein Eng Des Sel 22: 113–120. 10.1093/protein/gzn075 19074155 PMC2644406

[50] Wang K, Li M, Hakonarson H (2010) ANNOVAR: functional annotation of genetic variants from high-throughput sequencing data. Nucleic Acids Res 38: e164 10.1093/nar/gkq603 20601685 PMC2938201

